# Synthesis and Anthelmintic Activity of New Thiosemicarbazide Derivatives—A Preliminary Study

**DOI:** 10.3390/molecules25122770

**Published:** 2020-06-16

**Authors:** Katarzyna Dziduch, Przemysław Kołodziej, Agata Paneth, Anna Bogucka-Kocka, Monika Wujec

**Affiliations:** 1 Department of Organic Chemistry, Faculty of Pharmacy, Medical University of Lublin, 4A Chodzki Street, 20-093 Lublin, Poland; katarzynadziduch@o2.pl (K.D.); agata.paneth@umlub.pl (A.P.); 2 Chair and Department of Biology and Genetics, Faculty of Pharmacy, Medical University of Lublin, 4A Chodzki Street, 20-093 Lublin, Poland; przemyslawkolodziej@umlub.pl (P.K.); anna.kocka@umlub.pl (A.B.-K.)

**Keywords:** thiosemicarbazide, anthelmintic activity, intestinal helminths, *Rhabditis* sp.

## Abstract

Parasitic infections caused by different species of intestinal helminths still poses a threat to public health. There is a need to search for new, effective anthelmintic drugs. A series of novel thiosemicarbazides were synthesized and evaluated for their in vitro anthelmintic activity. The preliminary results showed that the most of synthesized compounds were very active. 4-Phenyl-1-[(1-methyl-4-nitroimidazol-2-yl)carbonyl]thiosemicarbazide and 4-(3-chlorophenyl)-1-[(1-methyl-4-nitroimidazol-2-yl)carbonyl]thiosemicarbazide showed a 100% mortality of nematodes and a high anthelmintic activity in both tested concentrations.

## 1. Introduction

Nothing could be further from the truth than that nowadays the problem of parasitic diseases caused by different species of intestinal helminths is no longer valid. Despite great knowledge of intestinal parasites and the fight against them, as well as the improvement in hygiene conditions, we can observe problems regarding parasitic diseases. One of the most common parasitic infections in humans are infections, caused by soil-transmitted helminths STH (soil-transmitted helminths also known as geohelminths). The World Health Organization (WHO) estimates that about 24% of the world’s population (1.5 billion people) are infected with soil-transmitted helminth infections worldwide. People are mainly affected by the roundworm, *Ascaris lumbricoides*; the whipworm, *Trichuris trichiura*; and the hookworms, *Necator americanus* and *Ancylostoma duodenale*. Intestinal helminths can cause, i.e., gastrointestinal infections, diarrhea, abdominal pain, loss of appetite, malnutrition, general malaise, weakness, dehydration, and weight loss. Other symptoms might include impairing the physical and mental growth of children [1,2,3,4,5].

Growing human migration, global travel, and climate change contribute to the increasing transmission dynamics of parasitic infections. The emergence of resistance to available drugs is also a major problem. Because of the emerging issue of parasitic infections and the limited number of drugs, as well as a small number of literature reports about the search for new compounds with anthelmintic activity, attempts have been made to obtain new compounds with a higher potency against parasitic nematodes.

There are relatively few reports in the literature about new research studies for antiparasitic compounds. One of the class of simple compounds that were evaluated for parasiticidal activity are thiosemicarbazones. These molecules were active against *Plasmodium falcipilarum*, *Trypanosoma equipedum*, *Trypanosoam crusi*, *Trypanosoma brucei,* and *Toxoplasma gondii* [6,7,8,9]. Thiosemicarbazides are very similar to thiosemicarbazones. A thiosemicarbazide skeleton is part of the structure of many compounds with interesting biological activities, including anticancer [10,11,12,13], antibacterial [12,13,14,15,16,17,18], antifungal [19], and analgesic [20].

In our laboratory, we obtained earlier and evaluated some thiosemicarbazides with good activity towards *T. gondii* [21,22]. Continuing in this direction, we designed and obtained thiosemicarbazide derivatives having a nitroimidazole substituent found in Metronidazole—the popular available antiparasitic drug.

## 2. Results and Discussion

### 2.1. Chemistry

**Scheme 1:**
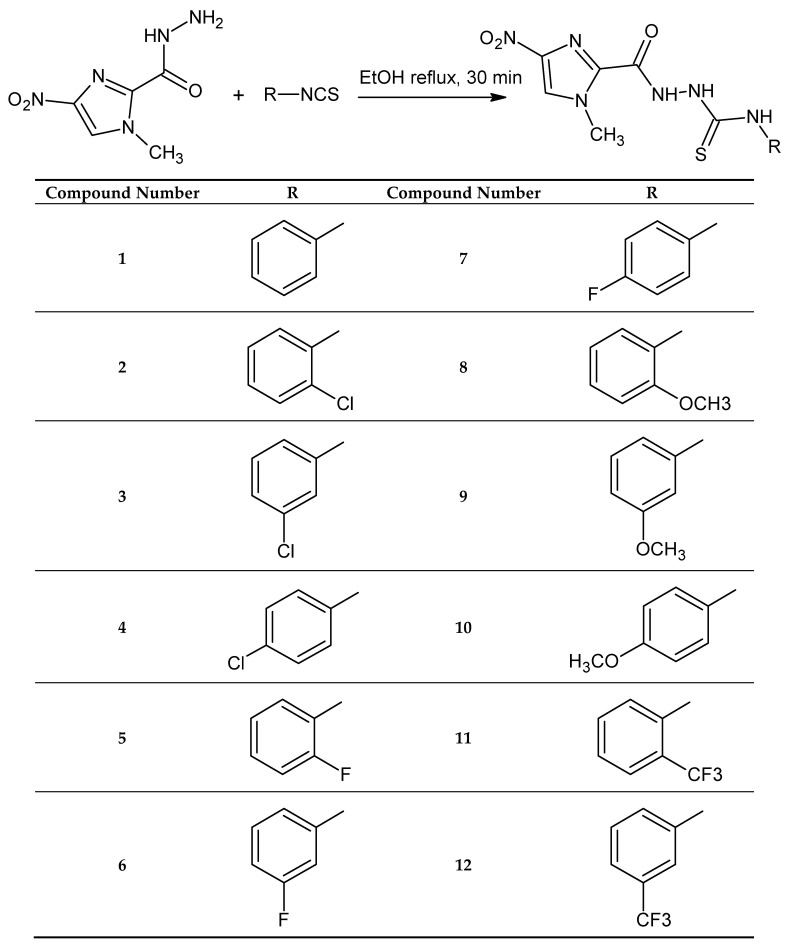
Synthetic route for the compounds **1**–**12**.

For the synthesis of new derivatives, 1-methyl-4-nitroimidazolole-2-carbohydrazide was used as a substrate by reacting it with selected isothiocyanates. The reactions were carried out by a known procedure by heating the substrates for 30 min in an ethanol medium [12,14,15]. Substituents for synthesis were selected in such a way that on the basis of the results of the biological studies, it was possible to determine the effect of the type of substituent on the anthelmintic activity. The new 1-[(1-methyl-4-nitroimidazol-2-yl)carbonyl]-4-substituted-thiosemicarbazides **1**–**12** were prepared according the Scheme 1.

### 2.2. Anthelmintic Activity

Most of the tested compounds showed anthelmintic activity. Among the tested thiosemicarbazide derivatives, the most active were derivatives **1**–**3**. The strongest activity was characteristic of 4-(2-chlorophenyl)-1-[(1-methyl-4-nitroimidazol-2-yl)carbonyl]thiosemicarbazide, which in both tested concentrations—i.e., 11.12 and 5.56 mg/mL—caused an excellent 100% mortality of nematodes of the genus *Rhabditis* sp. Both 4-phenyl-1-[(1-methyl-4-nitroimidazol-2-yl)carbonyl]thiosemicarbazide and 4-(3-chlorophenyl)-1-[(1-methyl-4-nitroimidazol-2-yl)carbonyl]thiosemicarbazide showed a 100% mortality of nematodes and high anthelmintic activity in both tested concentrations: 5.56 and 11.12 mg/mL. A slightly weaker anthelmintic activity was characterized by 4-(2-fluorophenyl)-1-[(1-methyl-4-nitroimidazol-2-yl)carbonyl]thiosemicarbazide **5** and 4-(3-fluorophenyl)-1-[(1-methyl-4-nitroimidazol-2-yl)carbonyl]thiosemicarbazide **6,** which at a concentration of 11.12 mg/mL induced a 100% mortality of parasites, and at a lower concentration of 5.56 mg/mL the survival of nematodes dropped to 4% in the case of the 3-fluorophenyl substituent and up to 32% for the 2-fluorophenyl substituent.

The next four compounds **7**, **10**, **11,** and **12** were then characterized by weaker anthelmintic activity. At neither of the two concentrations used was a 100% killing effect observed after 24 h of exposure to the compounds. At a concentration of 11.12 mg/mL, nematode survival was observed as follows: 2% for compound **12**, 8% in the case of compound **10**, 19% for compound **11**, and 39% for 4-fluorophenyl derivative **7**. At a concentration of 5.56 mg/mL, these values are 16%, 31%, 38%, and 63%, respectively.

The other three compounds demonstrated the weakest anthelmintic activity because at 5.56 mg/mL and 11.12 mg/mL they did not reach a 50% mortality of nematodes.

The percentage of nematode survival for the Albendazole used as reference medicine was 50% for the concentration 11.12 mg/mL (defined as the LC_50_—the median lethal concentration) and 59% for half of the former (5.56 mg/mL). It follows that the majority of the tested compounds showed a better performance at both concentrations than the control drug.

**Figure 1:**
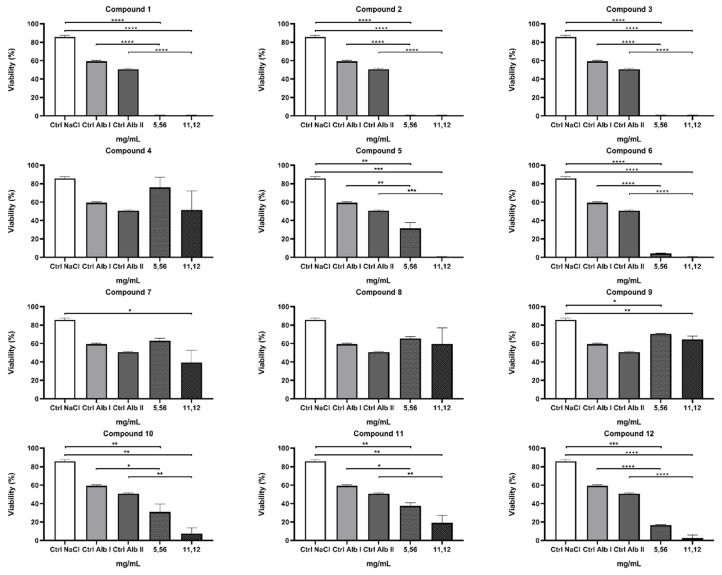
Viability (%) of *Rhabditis sp.* nematodes after 24 h of exposure in 2 experimental concentrations of the compounds tested. Ctrl—negative control; Ctrl Alb—positive control; albendazole I: 5.56 mg/mL; albendazole II: 11.12 mg/mL (*p* < 0.0001 ****, 0.0001 to 0.001 ***, 0.001 to 0.01 **, 0.01 to 0.05 *, ≥ 0.05 not significant).

**Figure 2:**
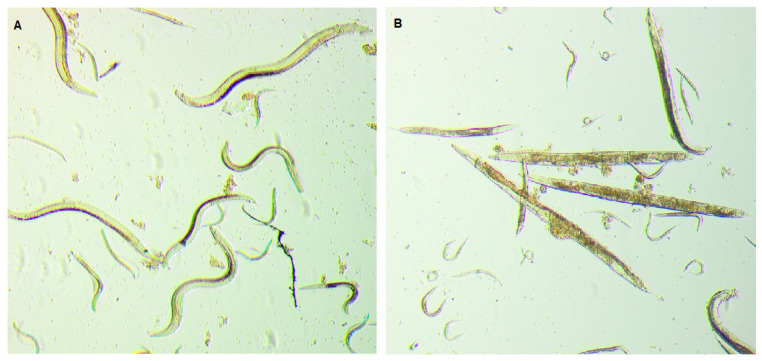
The nematode culture *Rhabditis* sp. (**A**) Control—alive; (**B**) after the action of the compound **5** (5.56 mg/mL)—dead.

The results of the anthelmintic activity are summarized in Figure 1 and Figure 2.

Based on the results obtained, it can be concluded that the presence of a halogen in the phenyl ring of thiosemicarbazide derivatives determines the activity. Derivatives of the electrodonating group (methoxy) were less active. In addition, the substitution position is very important. The substituent at the ortho or meta position of the phenyl ring increases activity, while para-substituent compounds were less active.

In our previous work, we obtained a series of thiosemicarbazide with promising activity against *Toxoplasma gondii*. Thus, we described the nematicidal effect of thiosemicarbazide. For the first time, we described tiosemicarbazides with excellent anthelmintic activity. Previously, the authors described the activity of thiosemicarbazide derivatives against earthworms (pontoscolex corethrusus); however, compounds at the concentration of 100 mg were inactive [23]. Other synthetic compounds were tested against *Rhabditis* sp., but no significant activity was observed. In 1995, Karouti synthesized new analogs of pyrantel. Two compounds were active against *Rhabditis pseudoelongata*. A derivative with a 2-hydroxyphenyl substituent was the most potent. Its activity was two times better than that of pyrantel, while the compound with a 2-tienyl substituent had a comparable activity with the reference drug [24]. 5-Fluorodeoxyuridine, an anticancer drug, was tested against nematode too. In long-range observations, it reduced the life span of *Rhabditidae tokai* [25]. 5-Nitrofurfural acetals were another group of the synthetic substances tested for anthelmintic activity. The best activity showed 5-nitrofurfural and its diacetate, whereas the dimethyl, diethyl, dipropyl, disopropyl, and dibutyl acetals were inactive [26].

Simple efficient synthesis and biological studies have made thiosemicarbazide derivatives potential candidates for drugs. The results obtained are preliminary and require further experiments to determine their molecular mechanism of nematicidal activity and determine the LC_50_ dose and in vivo studies.

## 3. Methods and Materials

All the substances used in this work were purchased from Sigma-Aldrich (Munich, Germany) and were used without further purification. The ^1^H-NMR spectra were recorded on the Bruker Avance 300 (Bruker BioSpin GmbH, Rheinstetten, Germany) in DMSO-*d*_6_ with tetramethylsilane as the internal standard. NMR spectra are presented in supplementary materials. The The IR spectra were recorded in KBr discs using a Nicolet 6700 FTIR spectrometer (Thermo Scientific, Waltham, MA, USA). The melting points were determined on a Fisher-Johns blocks melting point apparatus (Fisher Scientific, Schwerte, Germany) and are uncorrected. The purity of the compounds and progress of the reaction were monitored by TLC (aluminum sheet 60 F254 plates (Merck Co., Kenilworth, NJ, USA). We used the solvent system CHCl_3_/EtOH (10:1, *v*/*v*). Then elemental analyses were determined by a Perkin Elmer 2400 series II CHNS/O analyzer (Waltham, MA, USA), and the results were within ±0.4% of the theoretical value.

### 3.1. The Procedure for the Synthesis of 1-[(1-Methyl-4-nitroimidazol-2-yl)carbonyl]-4-substituted-thiosemicarbazide

A solution of 0.01 mole of 1-methyl-4-nitroimidazolole-2-carbohydrazide and an equimolar amount of appropriate isothiocyanate in 25 mL of anhydrous ethanol was heated under reflux for 30 min. Next, the solution was cooled and the solid formed was filtered off, washed with water and diethyl ether, dried, and crystallized from ethanol.

1-[(1-Methyl-4-nitroimidazol-2-yl)carbonyl]-4-phenyl-thiosemicarbazide **1**. Yield: 87%. m.p.: 175–177 °C. ^1^H-NMR (300 MHz, DMSO-*d*_6_) δ: 4.00 (s, 3H, CH_3_), 7.15−7.45 (m, 4H, Ar-H), 8.65 (s, 1H, CH), 9.80 (s, 2H, 2NH), 10.86 (s, 1H, NH). Anal. calc. for C_12_H_12_N_6_O_3_S (%): C 44.99; H 3.78; N 26.24. Found: C 44.95; H 4.02; N 26.28.

4-(2-Chlorophenyl)-1-[(1-methyl-4-nitroimidazol-2-yl)carbonyl]thiosemicarbazide **2.** Yield: 86%. m.p.: 180–183 °C. ^1^H-NMR (300 MHz, DMSO-*d*_6_) δ: 3.99 (s, 3H, CH_3_), 7.28–7.49 (m, 4H, Ar-H), 8.64 (s, 1H, CH), 9.60; 9.93; 10.98 (3s, 3H, 3NH). Anal. calc. for C_12_H_11_ClN_6_O_3_S (%): C 40.63; H 3.13; N 23.69. Found: C 40.85; H 3.30; N 23.63.

4-(3-Chlorophenyl)-1-[(1-methyl-4-nitroimidazol-2-yl)carbonyl]thiosemicarbazide **3.** Yield: 86%. m.p.: 190–193 °C. ^1^H-NMR (300 MHz, DMSO-*d*_6_) δ: 4.01 (s, 3H, CH_3_), 7.21–7.62 (m, 4H, Ar-H), 8.67 (s, 1H, CH), 9.78; 9.98; 10.91 (3s, 3H, 3NH). Anal. calc. for C_12_H_11_ClN_6_O_3_S (%): C 40.63; H 3.13; N 23.69. Found: C 40.83; H 3.28; N 23.65.

4-(4-Chlorophenyl)-1-[(1-methyl-4-nitroimidazol-2-yl)carbonyl]thiosemicarbazide **4.** Yield: 81%. m.p.: 195–197 °C. ^1^H-NMR (300 MHz, DMSO-*d*_6_) δ: 4.00 (s, 3H, CH_3_), 7.38–7.49 (m, 4H, Ar-H), 8.66 (s, 1H, CH), 9.76; 9.91 (2s, 2H, 2NH), 10.90 (s, 1H, NH). Anal. calc. for C12H11ClN6O3S (%): C 40.63; H 3.13; N 23.69. Found: C 40.78; H 3.23; N 23.70.

4-(2-Fluorophenyl)-1-[(1-methyl-4-nitroimidazol-2-yl)carbonyl]thiosemicarbazide **5.** Yield: 87%. m.p.: 192–195 °C. ^1^H-NMR (300 MHz, DMSO-*d*_6_) δ: 4.00 (s, 3H, CH_3_), 7.17–7.33 (m, 4H, Ar-H), 8.65 (s, 1H, CH), 9.56; 9.96; 10.97 (3s, 3H, 3NH). Anal. calc. for C_12_H_11_FN_6_O_3_S (%): C 42.60; H 3.28; N 24.84. Found: C 42.75; H 3.30; N 24.93.

4-(3-Fluorophenyl)-1-[(1-methyl-4-nitroimidazol-2-yl)carbonyl]thiosemicarbazide **6.** Yield: 80%. m.p.: 170–173 °C. ^1^H-NMR (300 MHz, DMSO-*d*_6_) δ: 4.02 (s, 3H, CH_3_), 6.97–7.49 (m, 4H, Ar-H), 8.65 (s, 1H, CH), 9.79; 9.96; 10.91 (3s, 3H, 3NH). Anal. calc. for C_12_H_11_FN_6_O_3_S (%): C 42.60; H 3.28; N 24.84. Found: C 42.73; H 3.34; N 24.95.

4-(4-Fluorophenyl)-1-[(1-methyl-4-nitroimidazol-2-yl)carbonyl]thiosemicarbazide **7.** Yield: 83%. m.p.: 193–196 °C. ^1^H-NMR (300 MHz, DMSO-*d*_6_) δ: 4.01 (s, 3H, CH_3_), 7.15–7.18 (m, 4H, Ar-H), 8.65 (s, 1H, CH), 9.72; 10.11; 10.88 (3s, 3H, 3NH). Anal. calc. for C_12_H_11_FN_6_O_3_S (%): C 42.60; H 3.28; N 24.84. Found: C 42.65; H 3.32; N 24.95.

4-(2-Methoxyphenyl)-1-[(1-methyl-4-nitroimidazol-2-yl)carbonyl]thiosemicarbazide **8.** Yield: 90%. m.p.: 210–212 °C. ^1^H-NMR (300 MHz, DMSO-*d*_6_) δ: 3.36 (s, 3H, CH_3_), 4.00 (s, 3H, CH_3_), 6.92–7.18 (m, 4H, Ar-H), 8.65 (s, 1H, CH), 9.19; 9.82; 10.96 (3s, 3H, 3NH). Anal. calc. for C_13_H_14_N_6_O_4_S (%): C 44.57; H 4.03; N 23.99. Found: C 44.74; H 4.20; N 24.05.

4-(3-Methoxyphenyl)-1-[(1-methyl-4-nitroimidazol-2-yl)carbonyl]thiosemicarbazide **9.** Yield: 85%. m.p.: 202–205 °C. ^1^H-NMR (300 MHz, DMSO-*d*_6_) δ: 3.74 (s, 3H, CH_3_), 4.00 (s, 3H, CH_3_), 6.73–7.24 (m, 4H, Ar-H), 8.65 (s, 1H, CH), 9.81(s, 2H, 2NH), 10.85 (s, 1H, NH). Anal. calc. for C_13_H_14_N_6_O_4_S (%): C 44.57; H 4.03; N 23.99. Found: C 44.76; H 4.18; N 24.07.

4-(4-Methoxyphenyl)-1-[(1-methyl-4-nitroimidazol-2-yl)carbonyl]thiosemicarbazide **10.** Yield: 83%. m.p.: 167–170 °C. ^1^H-NMR (300 MHz, DMSO-*d*_6_) δ: 3.75 (s, 3H, CH_3_), 4.01 (s, 3H, CH_3_), 6.89; 6.90 (d, 2H, Ar-H, *J* = 1.2 Hz), 7.28; 7.29 (d, 2H, Ar-H, *J* = 4.2 Hz), 8.64 (s, 1H, CH), 9.61; 9.71; 10.83 (3s, 3H, 3NH). Anal. calc. for C_13_H_14_N_6_O_4_S (%): C 44.57; H 4.03; N 23.99. Found: C 44.60; H 4.12; N 24.03.

4-(2-Trifluoromethylphenyl)-1-[(1-methyl-4-nitroimidazol-2-yl)carbonyl]thiosemicarbazide **11.** Yield: 83%. m.p.: 180–182 °C. ^1^H-NMR (300 MHz, DMSO-*d*_6_) δ: 3.99 (s, 3H, CH_3_), 7.36–7.72 (m, 4H, Ar-H), 8.64 (s, 1H, CH), 9.56; 9.93; 10.96 (3s, 3H, 3NH). Anal. calc. for C_13_H_11_F_3_N_6_O_3_S (%): C 40.21; H 2.86; N 21.64. Found: C 40.45; H 3.02; N 21.76.

4-(3-Trifluoromethylphenyl)-1-[(1-methyl-4-nitroimidazol-2-yl)carbonyl]thiosemicarbazide **12.** Yield: 89%. m.p.: 200–203 °C. ^1^H-NMR (300 MHz, DMSO-*d*_6_) δ: 4.02 (s, 3H, CH_3_), 7.50–7.85 (m, 4H, Ar-H), 8.67 (s, 1H, CH), 9.12; 10.05; 10.95 (3s, 3H, 3NH). Anal. calc. for C_13_H_11_F_3_N_6_O_3_S (%): C 40.21; H 2.86; N 21.64. Found: C 40.40; H 2.98; N 21.72.

### 3.2. Anthelmintic Activity Assay

The initial solutions were prepared at the following concentration: 100 mg/mL. Then, in order to obtain a homogeneous emulsion, sonication was carried out using an ultrasonic homogenizer (Hielscher Ultrasound Technology, Teltow, Germany).

The research involved the use of *Rhabditis* sp. nematodes, which live in soil in the natural environment. The culturing nematodes and research methodology of a nematocidal properties assessment was developed by the Department and Chair of Biology and Genetics of the Medical University of Lublin, Poland, patent no. 232918, Bogucka-Kocka A, Kołodziej P. The *Rhabditis* sp. nematodes were cultured in sterile 6-well plates on an agar medium enriched with bovine serum. After 4–5 days of culturing at room temperature, the growth and development of all the nematode development stages were observed.

*Rhabditis* sp. nematodes were eluted from the agar solid medium using 0.6% NaCl. They were then transferred to new sterile 24-well plates for culturing on a liquid medium. The tested compounds and albendazole were added to the culture prepared in 2 experimental concentrations: 5.56 mg/mL and 11.12 mg/mL. The respective new thiosemicarbazide derivatives were tested in a concentration corresponding to a survival rate of 50% for albendazole (LC_50_) and half of the former. The culture of nematodes without the addition of the tested compounds constituted the negative control, while the positive control was the culture with the addition of the following reference substance with anthelmintic activity: albendazole (Sigma, Munich, Germany).

After a 24 h exposure, the culture of *Rhabditis* sp. nematodes was observed in terms of development, deformity, damage, and motility using a stereoscopic and light microscope (Olympus, Tokyo, Japan). To determine the viability of nematodes, they were stained with methylene blue. The live and dead specimens were counted in counting chambers.

### 3.3. Statistical Analysis

A statistical analysis was performed using the GraphPad Prism 8 software (GraphPad Software, San Diego, CA, USA) using a one-way variance analysis ANOVA where statistically significant differences were adopted with the coefficient value of *p* < 0.0001 ****, 0.0001 to 0.001 ***, 0.001 to 0.01 **, 0.01 to 0.05 *, ≥ 0.05 not significant.

## 4. Conclusions

New thiosemicarbazide derivatives were synthesized and evaluated for their in vitro anthelmintic activity. Most of the compounds tested showed nematicidal activity. Selected compounds may become potential candidates for anthelmintic drugs. The most potent of them with phenyl **1**, ortho-chlorophenyl **2**, and meta-chlorophenyl **3** substituents were more active than albendazole. The results that have been obtained are preliminary and we will continue this project.

## Supplementary Materials

The following are available online, ^1^H-NMR spectra for compounds **1**–**12**.
