# Supplementary material for: Synthesis and Anthelmintic Activity of New Thiosemicarbazide Derivatives—A Preliminary Study
Source: Molecules. 2020 Jun 16;25(12):2770. doi: 10.3390/molecules25122770 (PMC7355614; doi:10.3390/molecules25122770)
Supplement: Supplementary file 1 [file molecules-25-02770-s001.pdf]

Supplementary information for

Synthesis and Anthelmintic Activity of New Thiosemicarbazide Derivatives – A Preliminary Study

Katarzyna Dziduch [1], Przemysław Kołodziej [2], Agata Paneth [1], Anna Bogucka-Kocka [2] and Monika Wujec [1,*],

[1] Department of Organic Chemistry, Faculty of Pharmacy, Medical University of Lublin, 4A Chodzki Street, 20-093 Lublin, Poland

[2] Chair and Department of Biology and Genetics, Faculty of Pharmacy, Medical University of Lublin, 4A Chodzki Street, 20-093 Lublin, Poland

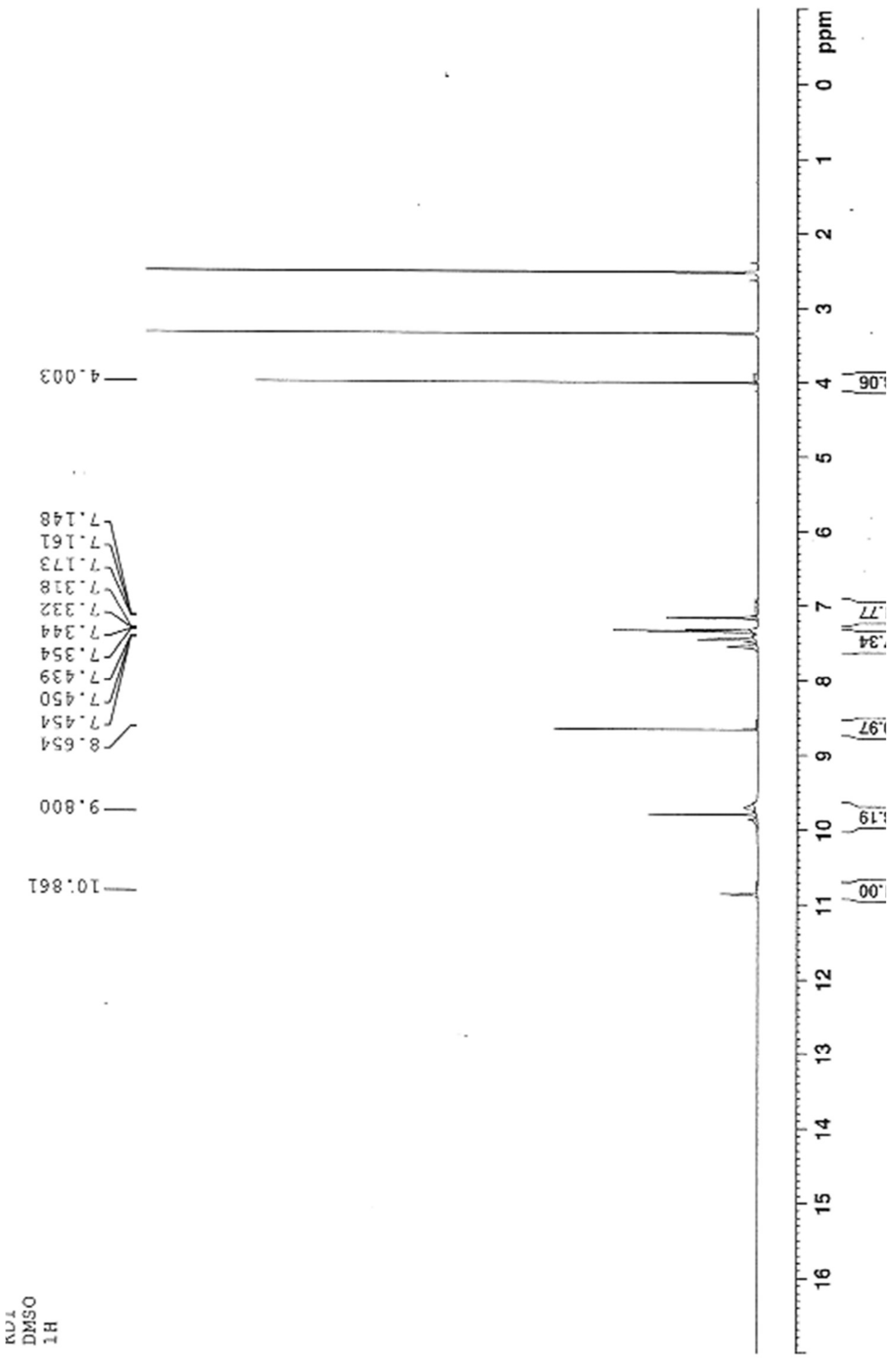
4.003
7.148
7.161
7.173
7.318
7.332
7.344
7.354
7.439
7.450
7.454
8.654
9.800
10.861
DMSO
1H
ppm

Compound 1.

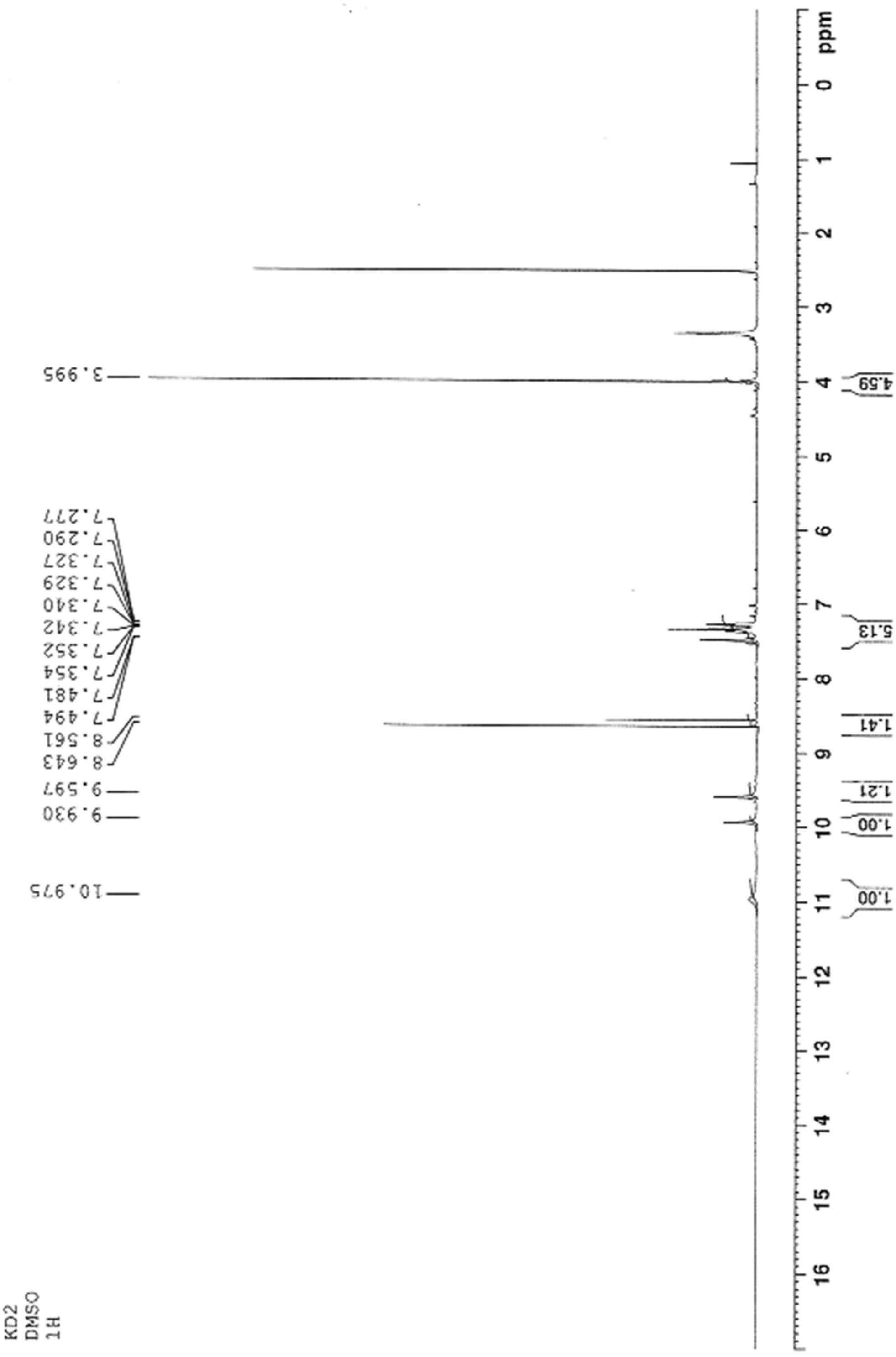
KD2
DMSO
1H
3.995
7.277
7.290
7.327
7.329
7.340
7.342
7.352
7.354
7.481
7.494
8.561
8.643
9.597
9.930
10.975
4.59
5.13
1.41
1.21
1.00
1.00
ppm

Compound 2.

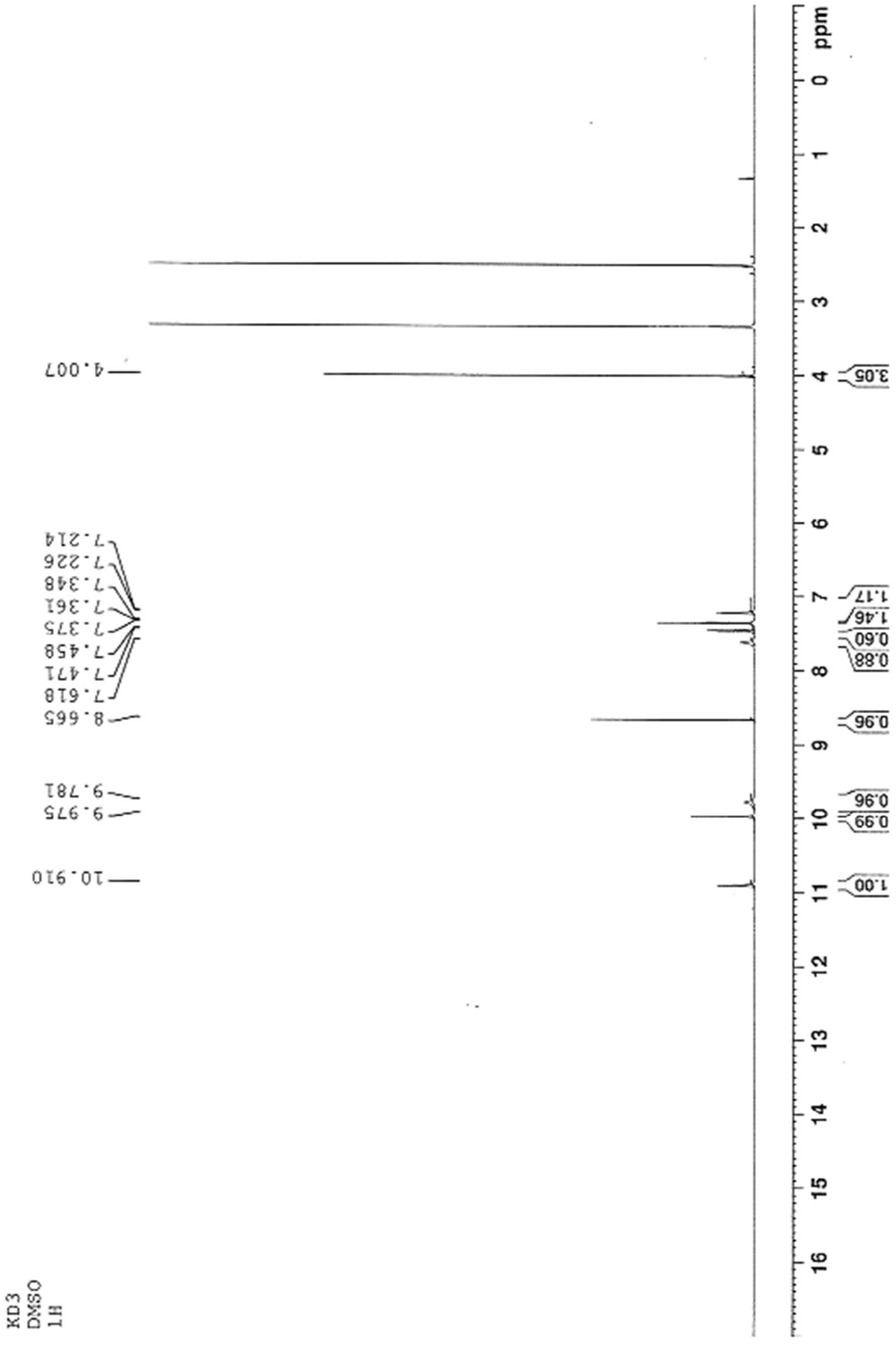
KD3
DMSO
1H
4.007
7.214
7.226
7.348
7.361
7.375
7.458
7.471
7.618
8.665
9.781
9.975
10.910
3.05
1.17
1.46
0.60
0.88
0.96
0.96
0.99
1.00
ppm

Compound 3.

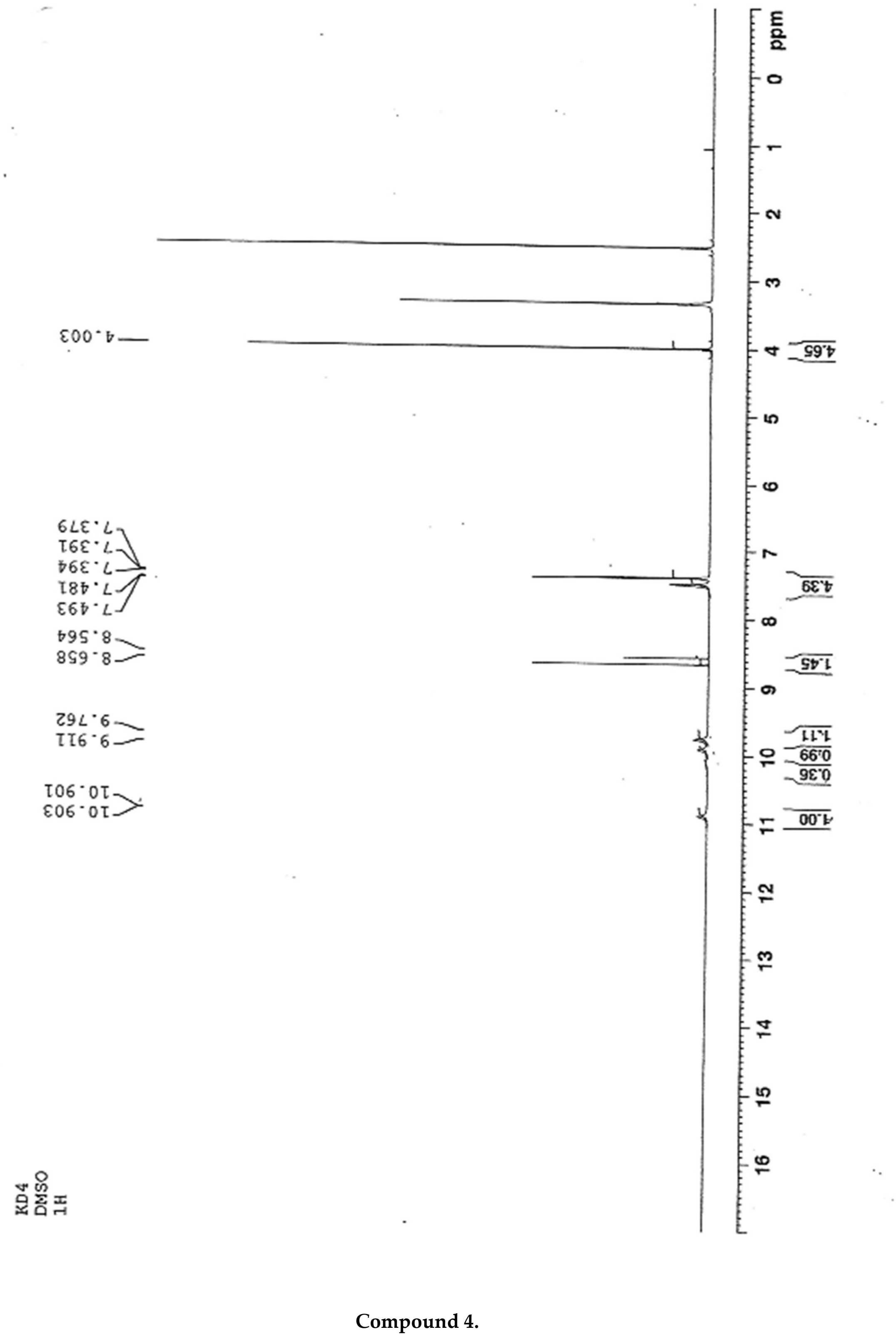
KD4
DMSO
1H
4.003
7.379
7.391
7.394
7.481
7.493
8.564
8.658
9.762
9.911
10.901
10.903
4.65
4.39
1.45
1.11
0.99
0.36
1.00
ppm

Compound 4.

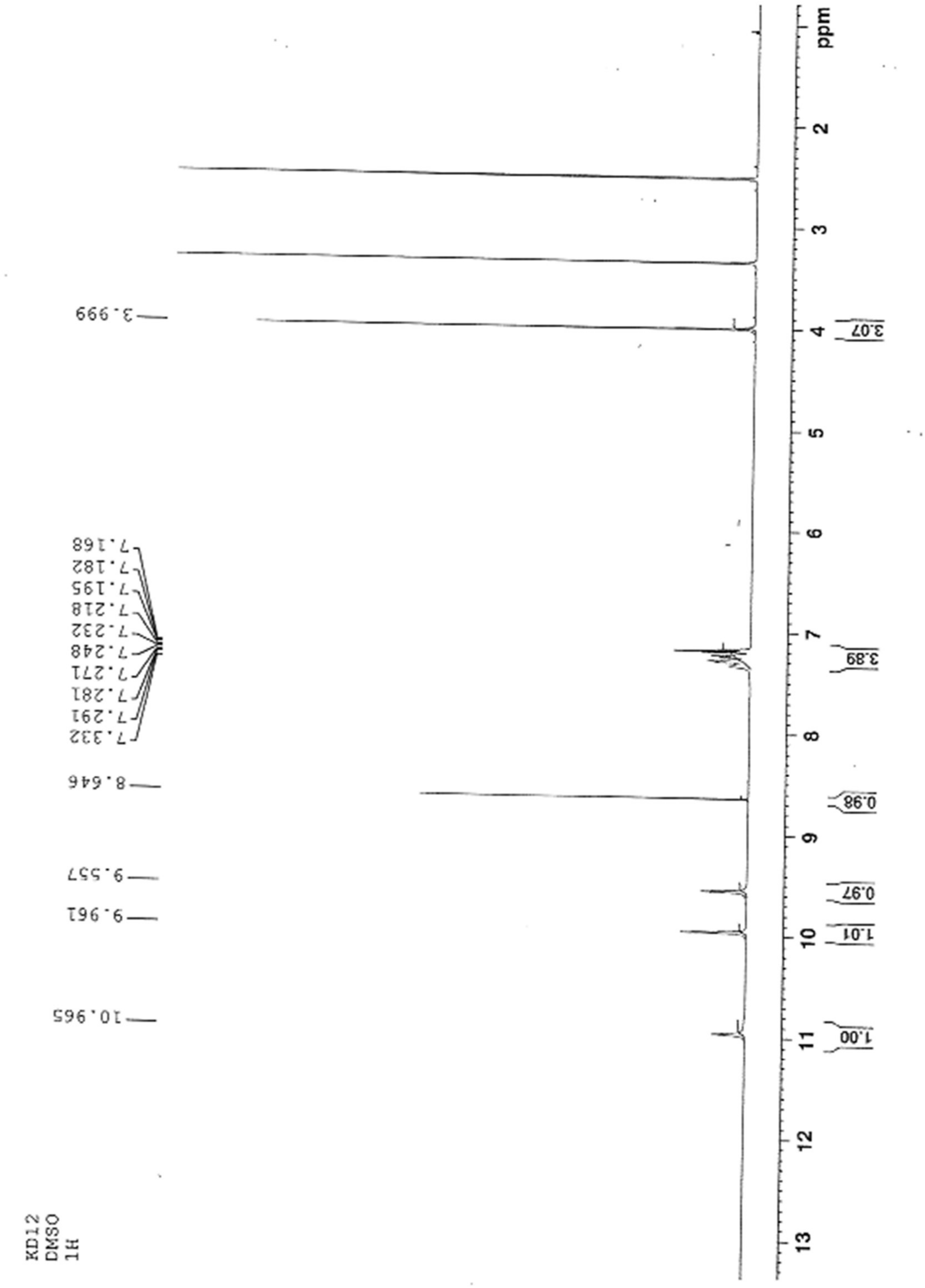
KD12
DMSO
1H
3.999
7.168
7.182
7.195
7.218
7.232
7.248
7.271
7.281
7.291
7.332
8.646
9.557
9.961
10.965
3.07
3.89
0.98
0.97
1.01
1.00
ppm

Compound 5.

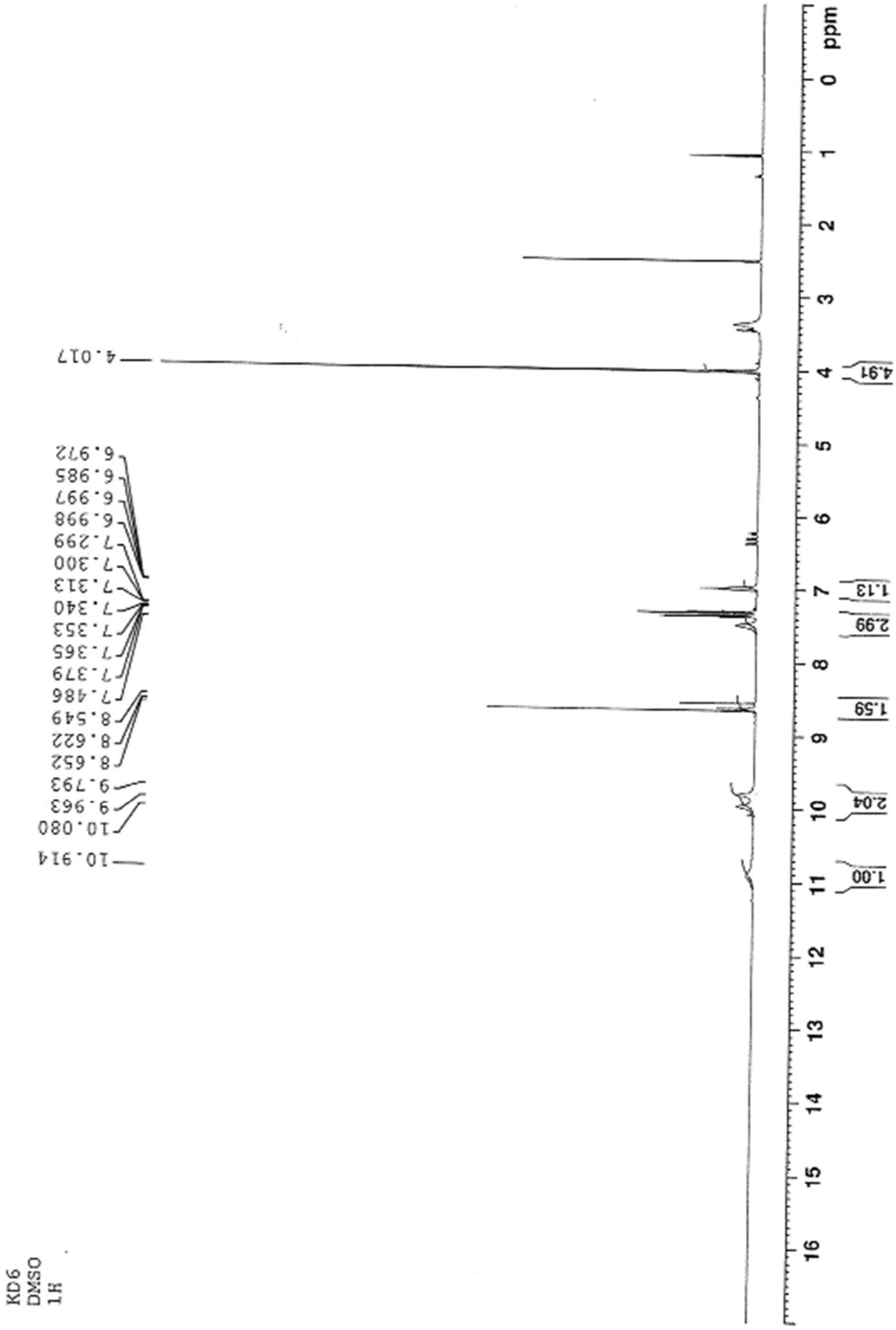
KD6
DMSO
1H
4.017
6.972
6.985
6.997
6.998
7.299
7.300
7.313
7.340
7.353
7.365
7.379
7.486
8.549
8.622
8.652
9.793
9.963
10.080
10.914
4.91
1.13
2.99
1.59
2.04
1.00
ppm

Compound 6.

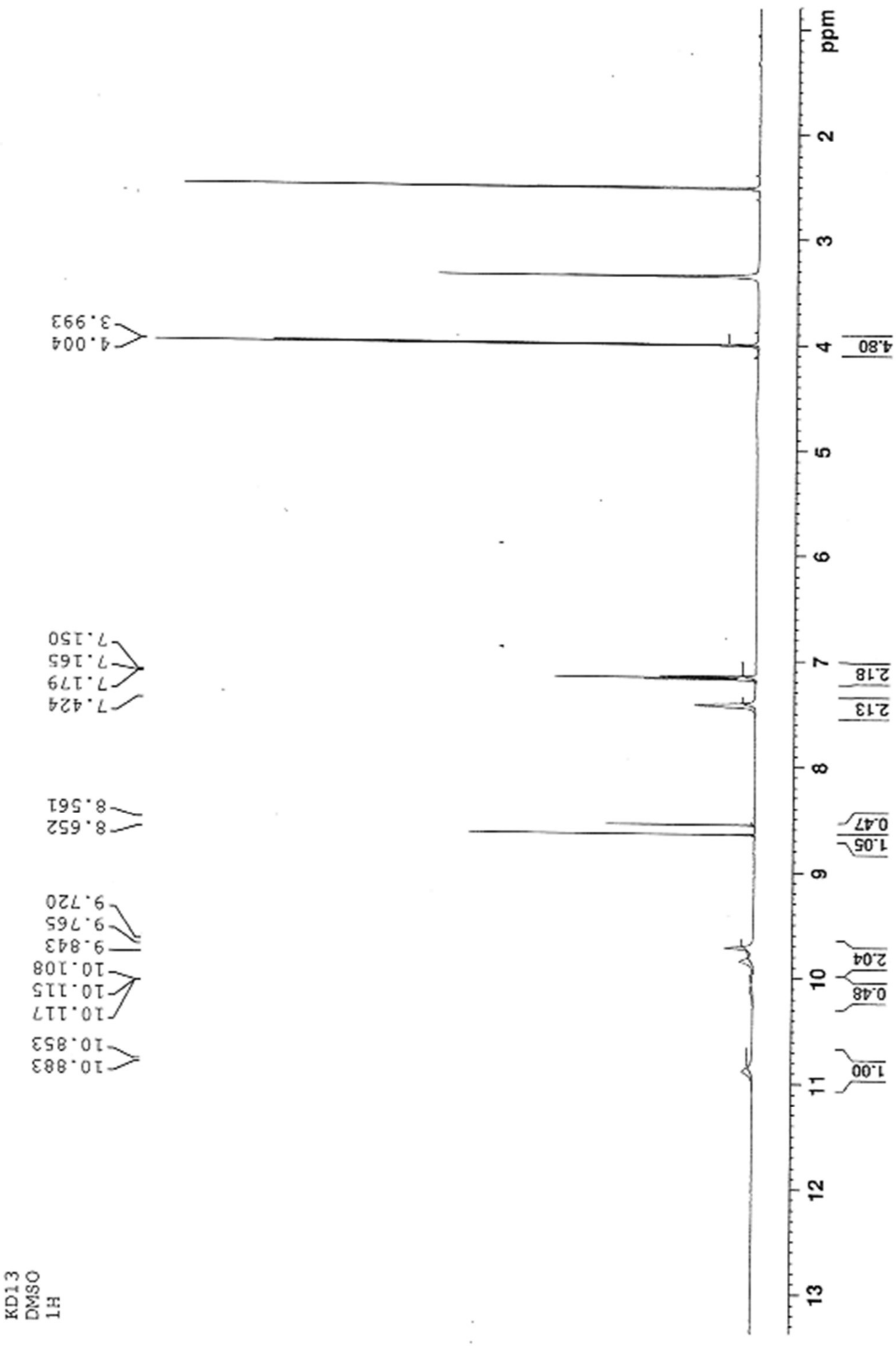
KD13
DMSO
1H
3.993
4.004
7.150
7.165
7.179
7.424
8.561
8.652
9.720
9.765
9.843
10.108
10.115
10.117
10.853
10.883
4.80
2.18
2.13
0.47
1.05
2.04
0.48
1.00
ppm

Compound 7.

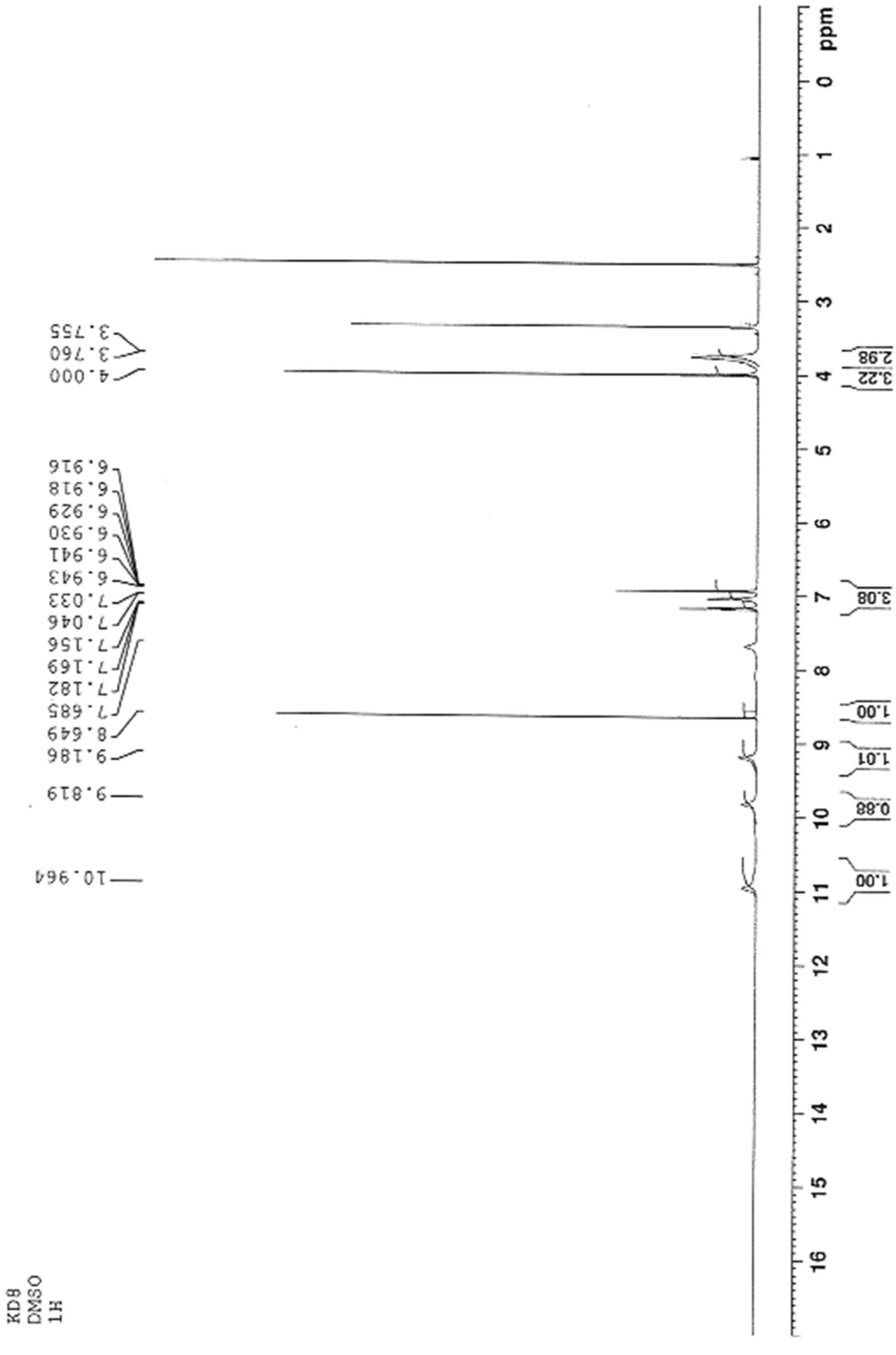
KD8
DMSO
1H
3.755
3.760
4.000
6.916
6.918
6.929
6.930
6.941
6.943
7.033
7.046
7.156
7.169
7.182
7.685
8.649
9.186
9.819
10.964
2.98
3.22
3.08
1.00
1.01
0.88
1.00
ppm

Compound 8.

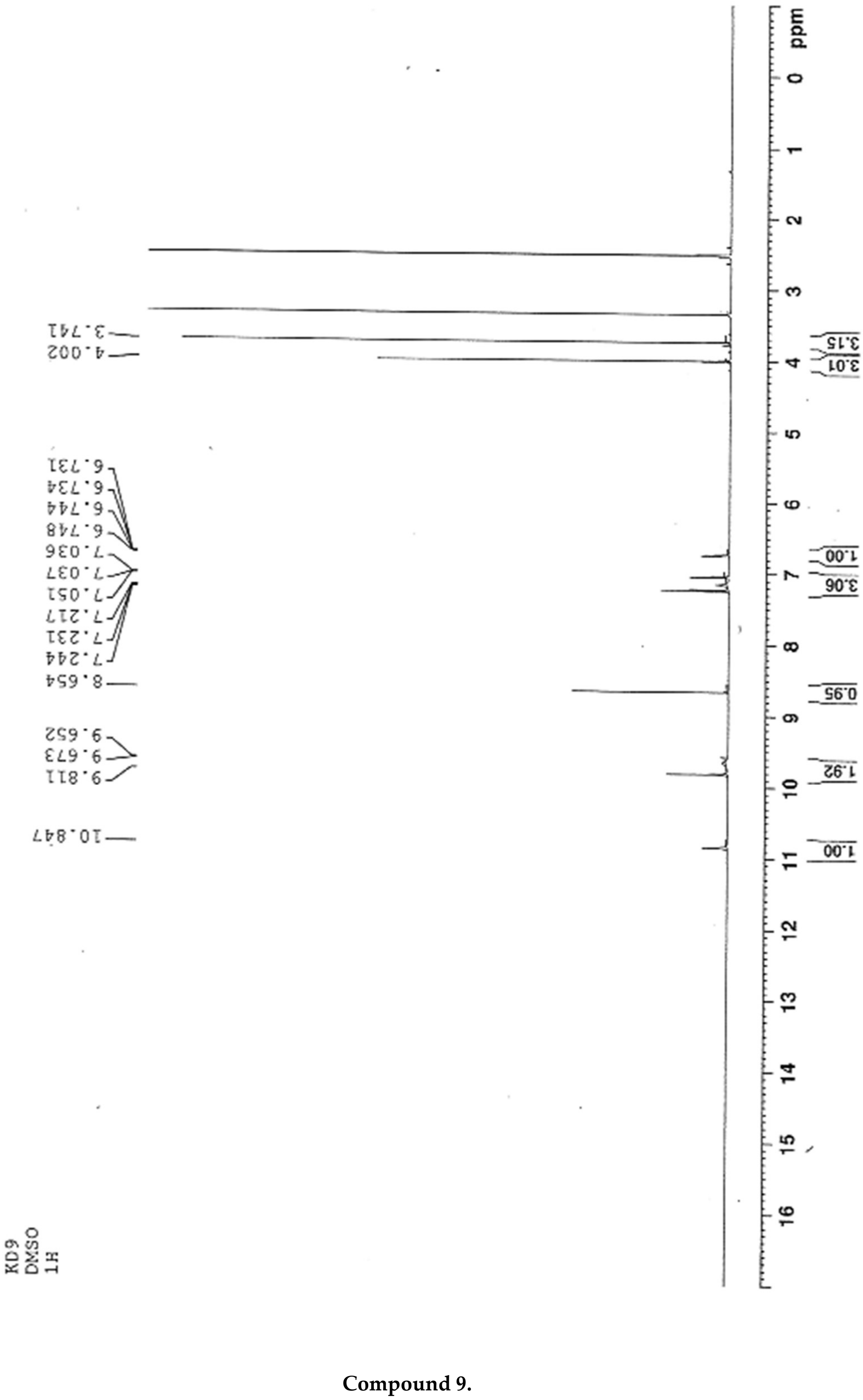
KD9
DMSO
1H
3.741
4.002
6.731
6.734
6.744
6.748
7.036
7.037
7.051
7.217
7.231
7.244
8.654
9.652
9.673
9.811
10.847
3.15
3.01
1.00
3.06
0.95
1.92
1.00
ppm

Compound 9.

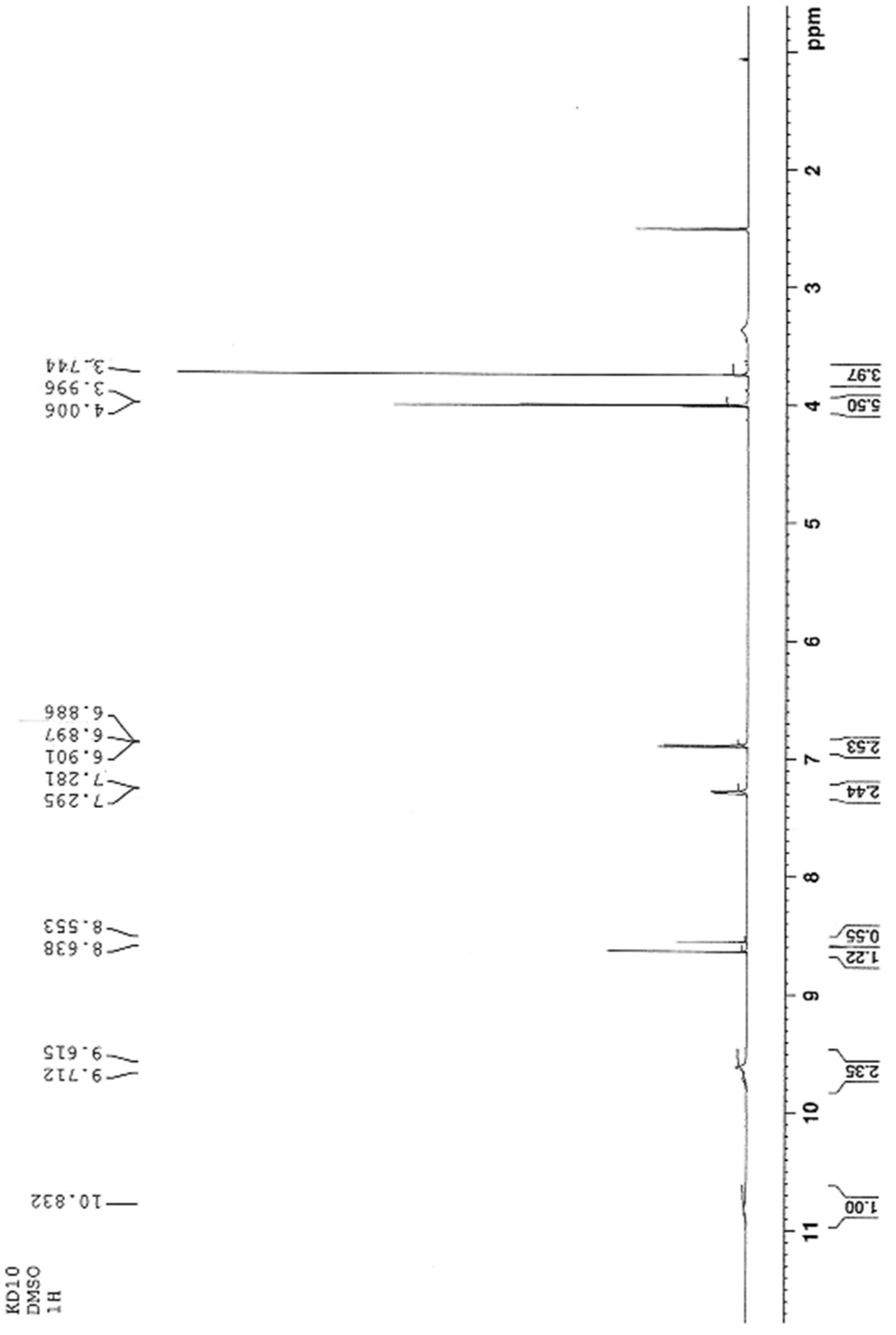
KD10
DMSO
1H
10.832
9.712
9.615
8.638
8.553
7.295
7.281
6.901
6.897
6.886
4.006
3.996
3.744
1.00
2.35
1.22
0.55
2.44
2.53
5.50
3.97
ppm

Compound 10.

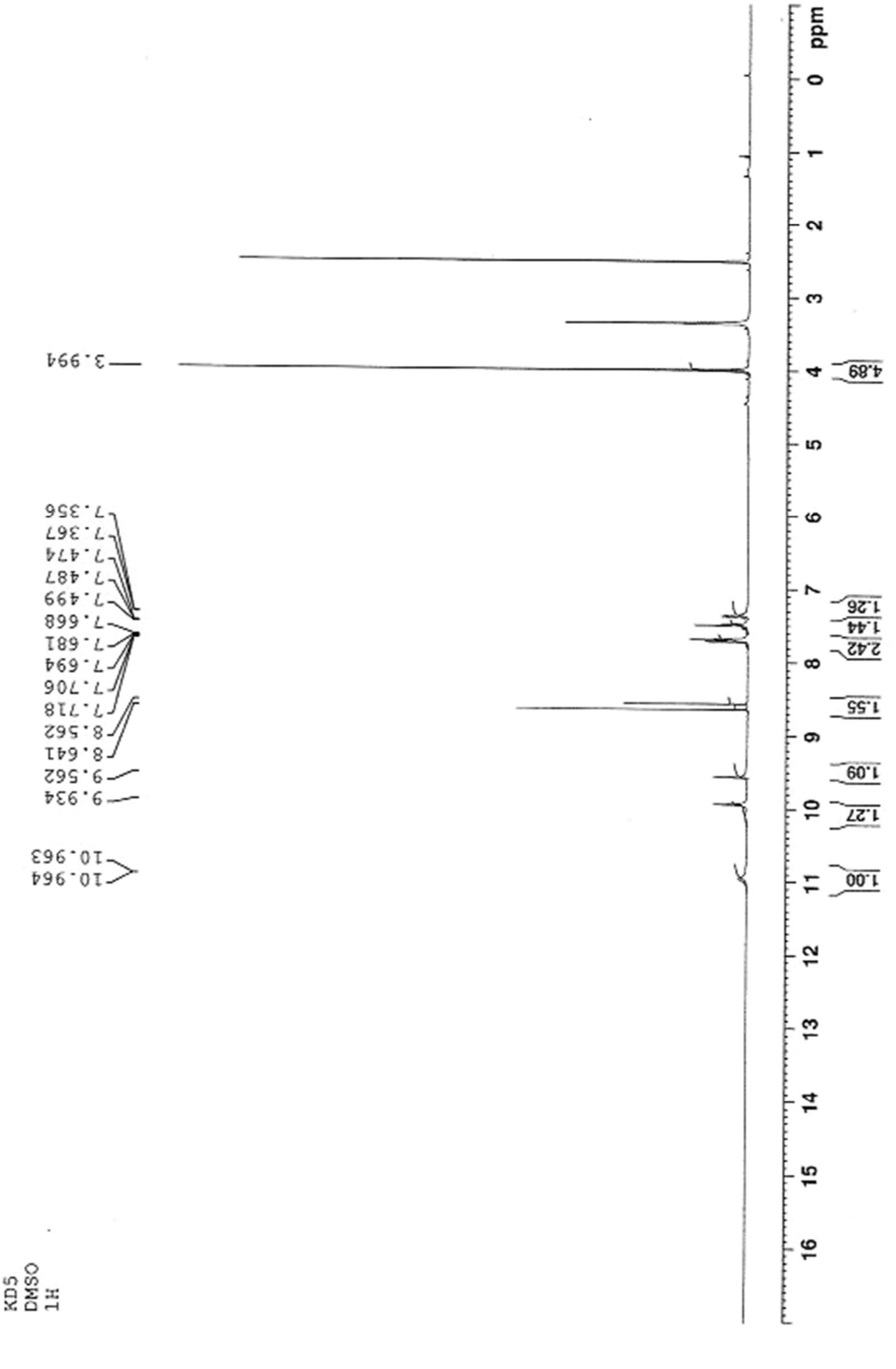
KD5
DMSO
1H
3.994
7.356
7.367
7.474
7.487
7.499
7.668
7.681
7.694
7.706
7.718
8.562
8.641
9.562
9.934
10.963
10.964
4.89
1.26
1.44
2.42
1.55
1.09
1.27
1.00
ppm

Compound 11.

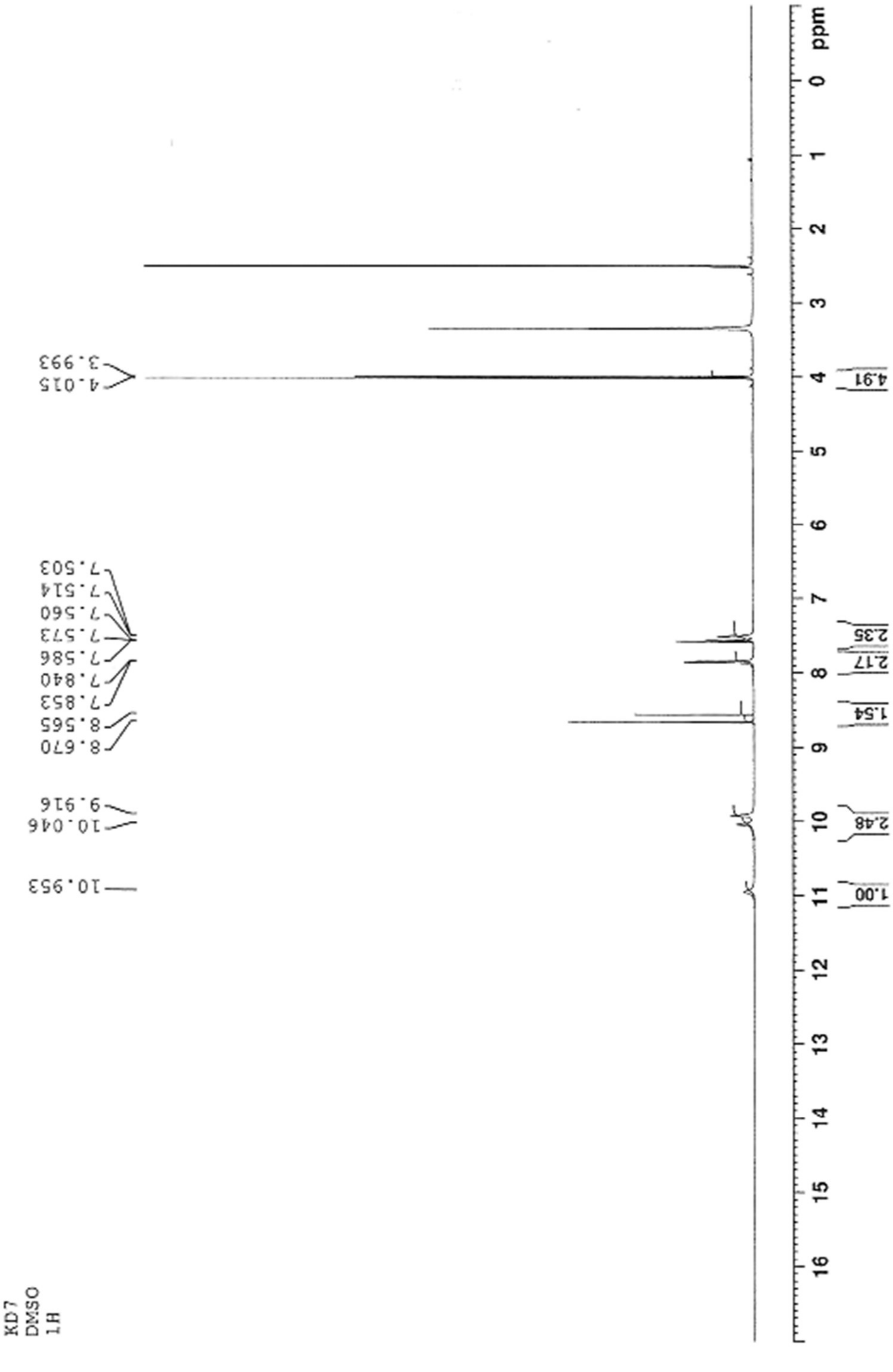
KD7
DMSO
1H
3.993
4.015
7.503
7.514
7.560
7.573
7.586
7.840
7.853
8.565
8.670
9.916
10.046
10.953
4.91
2.35
2.17
1.54
2.48
1.00
ppm

Compound 12.
